# Prevalence of an Intestinal ST40 *Enterococcus faecalis* over Other *E. faecalis* Strains in the Gut Environment of Mice Fed Different High Fat Diets

**DOI:** 10.3390/ijms21124330

**Published:** 2020-06-18

**Authors:** Beatriz Sánchez, Antonio Cobo, Marina Hidalgo, Ana M. Martínez-Rodríguez, Isabel Prieto, Antonio Gálvez, Magdalena Martínez-Cañamero

**Affiliations:** 1 Área de Microbiología, Departamento de Ciencias de la Salud, Universidad de Jaén, Paraje de Las Lagunillas s/n, 23071 Jaén, Spain; bsa00002@red.ujaen.es (B.S.); acmolino@ujaen.es (A.C.); mhidalgo@ujaen.es (M.H.); agalvez@ujaen.es (A.G.); 2 Departamento de Estadística e Investigación Operativa, Universidad de Jaén, Paraje de Las Lagunillas s/n, 23071 Jaén, Spain; ammartin@ujaen.es; 3 Área de Fisiología, Departamento de Ciencias de la Salud, Universidad de Jaén, Paraje de Las Lagunillas s/n, 23071 Jaén, Spain; iprieto@ujaen.es

**Keywords:** *E. faecalis*, MLST, proteomics, olive oil, high fat diets

## Abstract

*E. faecalis* is a commensal bacterium with specific strains involved in opportunistic and nosocomial infections. Therefore, it is important to know how the strains of this species are selected in the gut. In this study, fifteen *E. faecalis* strains, isolated over twelve weeks from the faeces of mice fed standard chow or one of three high fat diets enriched with extra virgin olive oil, refined olive oil or butter were subjected to a genetic “Multilocus Sequence Typing” study that revealed the presence of mainly two genotypes, ST9 and ST40, the latter one prevailing at the end of the research. A V3–V5 sequence comparison of the predominant ST40 strain (12B3-5) in a metagenomic study showed that this sequence was the only *E. faecalis* present in the mouse cohort after twelve weeks. The strain was subjected to a comparative proteomic study with a ST9 strain by 2D electrophoresis and mass spectrometry. After comparing the results with a *E. faecalis* database, unshared entries were compared and 12B3-5 showed higher antimicrobial production as well as greater protection from environmental factors such as xenobiotics, oxidative stress and metabolite accumulation, which could be the reason for its ability to outcompete other possible rivals in an intestinal niche.

## 1. Introduction

During recent years, there have been a growing number of studies on the effect of diet on the composition of the intestinal microbiota, and the possible influence of a microbial imbalance or dysbiosis on animal physiology has been widely recognized [1]. The study of the microbiota has become an important factor to be considered in this science, since changes in the microbiota could presumably have a concomitant effect on the host organism [2], even more so if we take into account that the gut microbiota is composed of a large number of taxa with complex ecological relationships among them as well as with a number of metabolite exchanges with the host.

Within this field of research, there is a clear interest in diets enriched in fat, due to the negative outcomes they cause on health. Several studies have reported that high-fat diets alter the intestinal microbiota in mice [3,4,5]. Different high-fat diets (HFD) have different impacts on the Bacteroidetes/Firmicutes ratio, and an interesting aspect is the influence of an HFD on the change in the faecal microbiota in hyperlipidemic mice with this ratio increased [6]. Remarkably, an alpha-linolenic acid-rich diet lowers the Bacteroidetes/Firmicutes ratio [7]. Recent studies reveal that a diet enriched with extra virgin olive oil (EVOO) has a distinctive effect on the intestinal microbiome compared to a butter enriched diet and that this effect is related to the physiological benefits exerted by EVOO [8,9]. In these studies, EVOO was used as an example of unsaturated fat in the diet. However, it is important to consider that a large part of the population consumes olive oil after it has been subjected to a purifying process that not only affects its organoleptic characteristics, softening its taste, but also eliminates polyphenols and other minority components essential for the exertion of these benefits [10]. That is why refined olive oil (ROO) was also included in another subsequent study [11]. In all these reports, clear differences were revealed both in the profile of the faecal microbiota and in several physiological variables related to the metabolic syndrome. Some of these variables maintain statistically significant correlations with the percentages of several taxa that were increased in the group fed a diet rich in butter but not in the group under a diet rich in EVOO [8,11]. The microbiota from mice fed refined olive oil scored differently than those from the EVOO group in half of the bacterial taxonomical families with statistically significant differences among the diets [11]. These results are clearly indicative of a connection between certain foods, some physiological factors and the occurrence of specific taxa, but they also reinforce the proposal that polyphenols and other unsaponifiable EVOO components are involved in some of the effects of this fat by means of the modulation of the gut microbiota [11].

However, these new techniques can only address global taxa, without providing discriminatory data on how different strains of a particular bacterial taxon may develop in response to the diet. Still, changes at the strain level are probably the first ones to occur in the intestine.

One of the best studied genera in contamination through the oral–faecal route is the genus *Enterococcus*. Enterococci are frequently present in foods and fermentations, but they also have a growing role as nosocomial pathogens, so understanding their epidemiology and population structure has become more and more essential, and many of their virulence and resilience factors are already well known [12]. Therefore, it constitutes a good model to study how the type of nourishment can influence the intestinal prevalence of the different strains and, in this way, how certain diets may or may not protect the host, promoting or hindering the growth of strains with different levels of virulence, once they have reached the intestine. Since we know the antimicrobial effect of olive oil on different bacterial taxa both in vitro [13] and in a murine model [11], it seemed interesting to evaluate its possible role in the selection of the strains that will thrive in the gut of the host, when this fat is included in the diet.

Consequently, in another subsequent study, the safety level of enterococcal strains isolated from the intestinal microbiota of mice fed a standard diet or one enriched with butter and virgin and refined olive oil was addressed, by assessing the resistance of the strains to antibiotics, virulence factors and the production of biogenic amines, in order to ultimately determine if there are statistically significant differences among four groups of enterococci isolated from those four different diets. Thus, as also reported in [14], we found statistically significant differences among the diets in the percentage of antibiotic resistance and in the presence of the enterococcal surface protein gene (*esp*). When the resistance of the strains to virgin or refined olive oil was studied, only the group of enterococci from the mice fed the high fat diets showed a significantly higher percentage of resistance to refined olive oil, while both types of oil equally inhibited those isolated from mice under the standard diet [14]. Additionally, we had also been unable to isolate any strain from EVOO-fed mice at the end of the experimental period. In conclusion, in our study, the type of diet of the host had a significant influence on the prevalence of certain attributes of the strains that thrived within.

In the present report, we have gone a step further and focused on studying the enterococcal species most frequently isolated in the previously described experiment, *Enterococcus faecalis*. *E. faecalis* population structures have repeatedly been studied using multilocus sequencing typing (MLST), and some clonal complexes have been found to be enriched among clinical strains, while commensal isolates are more frequently found in others [15]. Following this epidemiology-oriented investigation, much effort has been taken to evaluate the safety of the *E. faecalis* strains that can be found in different types of food [16,17,18]. However, to our knowledge, nothing has been done on how the intake of different foods may influence the safety of the prevalent *E. faecalis* strains in the host intestines, contributing to or preventing certain clinical isolates from spreading in the host community. In this work, we present a preliminary study on this subject.

## 2. Results

### 2.1. Strain Distribution in Diets and at Timepoints

All the *E. faecalis* strains detected in a wider enterococci collection [14] were selected for this study, numbering, in total, fifteen strains. The enterococci collection had been isolated from mice fed on different diets. Out of the fifteen *E. faecalis* strains, five were isolated at the beginning of the study, with the following distribution: one from the butter fed group (BT), two from the refined olive oil fed group (ROO), one from the virgin olive oil fed group (EVOO) and one from the group fed on the standard diet (SD). Seven strains were isolated after six weeks of diets (two from SD, one from BT, three from ROO and one from EVOO). Finally, three strains were isolated at the end of the experiment, after 12 weeks, all of them from the butter fed group of mice. 

### 2.2. Bacteriocin Production

When assaying the bacteriocin production both with the spot-on-a-lawn and the agar well methods, four strains showed a small halo of 1 mm around the well diameter. Two of them were active against *S. aureus* (0S1-1 and 12B1-4); one, against *E. faecalis* (6B5-1); and one was active against both *S. aureus* and *Listeria* (12B3-5). None were active against *E. coli* or *Salmonella*.

### 2.3. Multilocus Sequence Typing (MLST) Analysis

**Figure 1:**
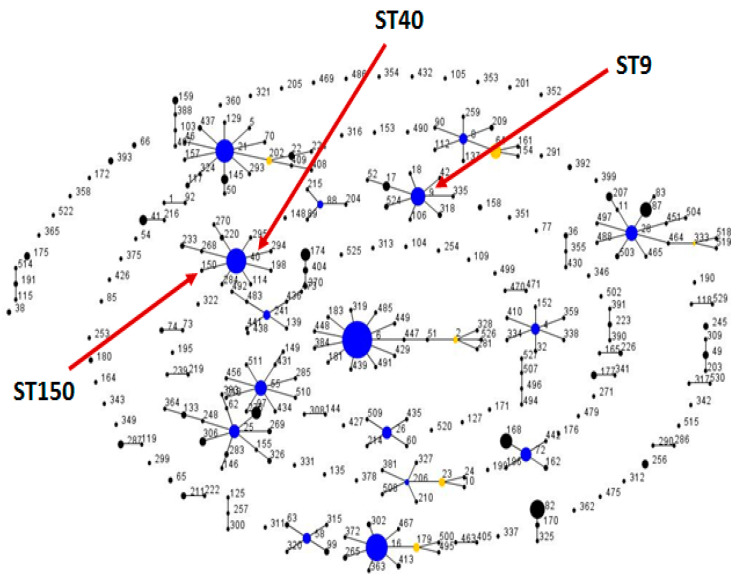
Population snapshot of *Escherichia faecalis* with the entire multilocus sequencing typing (MLST) database in a single eBURST diagram. STs differing in one allele are connected. Colours indicate predicted primary (blue) and secondary (yellow) founders. The areas of each of the circles indicate the prevalence of the ST in the input data. STs 9, 40 and 150 are marked by arrows.

Table 1 shows the allelic profile for each strain and the sequence type (STs) that this allele combination determines. The closest match for each strain in a BLAST search was also included in the table, with a 100% identity in all cases. According to these results, all strains belong mostly to two sequence types, ST9 and ST40, corresponding to two BLAST matching strains, *E. faecalis* KB1 and D32, respectively. Only in one case is there an additional type, ST150 (6B2-1), although its BLAST match corresponds again to *E. faecalis* D32. Figure 1 shows an *E. faecalis* eBURST diagram where the three sequence types detected are shown. At the beginning of the experiment, before high fat diets were applied, both ST9 and ST40 are present (ST40 in the BT and ROO diets, while ST9 is in SD, EVOO and also ROO). However, once high fat diets are applied, only ST40 is isolated (except the very close, above-mentioned case of ST150). At the end of the experiment, after twelve weeks of diets, *E. faecalis* could only be isolated from butter-fed mice and, again, all strains were ST40. 

### 2.4. r16S V3-V5 Sequence Identification and Analysis

In order to identify these STs in the metagenomic data available from our previous reports, the V3–V5 region of the r16S gene of strain 12B3-5 was sequenced so that we were able to align the resulting sequence with the sequences previously obtained for *E. faecalis* by pyrosequencing [8,11]. Table 2 shows the results obtained when comparing 12B3-5 with all the *E. faecalis* sequences present in the metagenomic study.

As indicated in Table 2, half of the copies detected in faecal samples from butter-fed mice at 0 weeks were identical to 12B3-5 and they were restricted to one mouse. However, after 12 weeks of experiment, all the sequences detected were identical, matched our strain and were found in five out of nine mice. This did not happen in the other diets were *E. faecalis* was detected only in two mice, one of the EVOO group with two sequences and one from the ROO group with three, and none of them matched 12B3-5. The SD diet did not present any *E. faecalis* sequence. 

**Figure 2:**
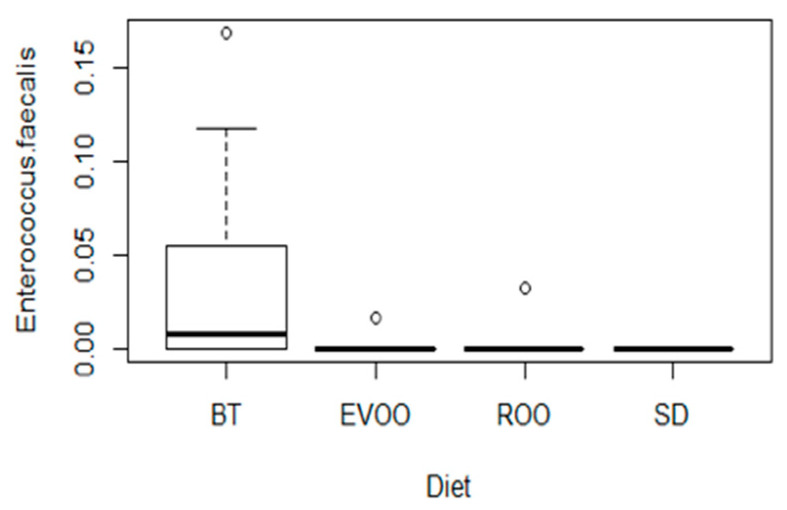
Box-plot representation of the percentage distribution of *E. faecalis* metagenomic sequences among faecal samples from mice fed a diet enriched with butter (BT; *n* = 9), virgin olive oil (EVOO; *n* = 9), refined olive oil (ROO; *n* = 9) or standard chow (SD; *n* = 8) (*p* = 0.014).

All the metagenomic sequences annotated as *E. faecalis* at the end of the experiment were subjected to a Kruskal–Wallis analysis to confirm the higher presence in the butter-fed group of mice, and the difference was clearly significant (*p* = 0.014), with post-hoc pairwise comparisons showing significance between BT and SD (*p* = 0.025). Figure 2 shows the box plot representation of the percentage of sequences of *E. faecalis* in the four groups.

### 2.5. Proteomic Studies

Given the interest raised by the two STs detected and the high prevalence of one of them in the metagenomic results of the mice faeces, two strains (12B3-5 and 0V5-2) were subjected to further analysis in order to compare their proteomes. 12B3-5 was chosen as an example of ST40, while 0V5-2 was selected as a representative of ST9.

**Figure 3:**
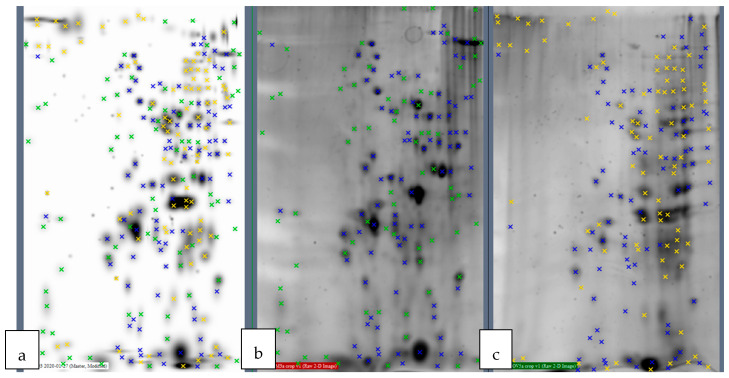
Spot comparison after 2D gel electrophoresis using the PDQuest^TM^ 8.0.1. Analysis Software (Bio-Rad, Hercules, CA, USA). **(a)** Both gels overlapped; (**b**) Gel corresponding to 12B3-5; **(c)** Gel corresponding to 0V5-2. Green crosses show 12B3-5-specific spots; yellow crosses show 0V5-2-specific spots; blue crosses show spots that are common to both samples.

Protein extracts were obtained from overnight cultures under the same growth conditions, and 2D gel electrophoresis images were obtained. As shown in Figure 3, the spot distribution was overall similar but clear differences could be appreciated between both extracts as marked in the figure.

This result was decisive in proceeding to further analyse the samples by Orbitrap Mass Spectrometry and contrast the obtained profiles with the proteomic *E. faecalis* Uniprot.org database. As a consequence, we obtained two very similar files, and the differences between them were searched for. The file corresponding to the proteome digestion of strain 0V5-2 contained 37,910 peptides corresponding to 7708 proteins. In the sample of strain 8M3-5, 32,333 peptides were obtained, corresponding to 6431 proteins. When comparing the archives of the two strains, 280 proteins were detected, which were present in only one strain, not being found in the other one (Supplementary Materials S1). Many of these proteins are variations of others already existing in the opposite strain. However, for other specific proteins, no similar entry could be found at all. Specific proteins were grouped according to their function as found in the available literature, and ten categories were designed. Table 3 shows the percentage in number of such proteins of each of the strains included in every group.

## 3. Discussion

A large collection of enterococci isolated from faeces of mice fed with different high fat diets enriched in butter, virgin olive oil and refined olive oil and from mice fed with standard chow [8,11] was screened for *E. faecalis* strains, and fifteen of them were uncovered [14] and further studied in the present report. The objective of this work is to provide basic knowledge on how different strains of the same intestinal species can thrive and be selected in a controlled population and if a specific diet may have an influence on certain strains or traits. Additionally, this information becomes more interesting when dealing with a bacterium (*E. faecalis*) well known for its commensal and environmental prevalence but also for the increasing role that certain genotypes have in opportunistic and nosocomial infections.

These strains had been subjected to Randomly Amplified Polymorphic DNA (RAPD) analysis to ensure they were genetically different [14]. However, when undergoing MLST analysis as shown above, all of them belonged to only three sequence types: ST9, ST40 and ST150 (one strain). A BLAST analysis linked all the ST9 isolates with *E. faecalis* KB1 (100% identity), while the ST40 and ST150 isolates, closely related to each other, were 100% identical to *E. faecalis* D32. The different STs were never isolated from the same animal at the same timepoint, even though a search through our metagenomic database uncovered different *E. faecalis* sequences in one butter-fed mouse at the beginning of the experiment, half of them with a 100% coincidence with the V3–V5 sequence of the ST40 strain 12B3-5. In spite of this variability at the start point, this V3–V5 sequence was the only one found among the reads in the metagenomic data of the butter-fed animals at the end of the experiment. 

Even though commensal and clinical *E. faecalis* isolates do not appear to be as evolutionary distinct as those in *E. faecium*, in both species, certain clonal complexes (CCs) appear to be significantly associated with hospital-derived isolates [12]. However, in the case of *E. faecalis*, CCs contain both clinical and commensal isolates and the incidence as opportunistic invaders varies across different geographical regions. In spite of this, certain sequence types seem to be more widespread than others in the reports of nosocomial outbreaks. Initially, CC9, the clonal complex where ST9 clusters, was reported to be enriched among hospital-derived strains [15] with a high presence of antibiotic resistance [19]. However, in later years, studies on the recently isolated strains in Europe uncovered the epidemiological success of other CCs, displacing CC9 as a potentially risky lineage [20] and also reporting CC9 as a commensal isolated from porcine milk [21] or migrating birds [22]. Regarding ST40, which was previously described as the most frequent ST according to a large collection [17], it was defined as an emerging epidemic clone, probably the most prevalent in Central Europe [23], and its presence in epidemiological reports has been maintained across Europe [24,25] and elsewhere [26]. More recently, a comprehensive analysis of 42 ST40 isolates from different sources and geographical origins [27], including a D32 strain whose genome had been fully sequenced [28], gave new information about this sequence type. According to these authors, there is a high level of similarity throughout the collection, and the study of their differences indicated that recombination within the clonal lineage was minor. Differences between strains from a commensal or clinical background, or animal or human source, were not found. In addition, authors showed that the strain D32 had greater capacity for adherence to human cell lines, higher pathogenic potential and faster growth in a murine bacteraemia model compared to a non-D32 ST40.

In our case, differences have not been found between the two sets of strains in either sequence type, neither with respect to antibiotic resistance, virulence factors or biogenic amine production [14] nor bacteriocin production in this report (Section 2.2). In spite of this, specifically, 12B3-5 is the only strain to show antimicrobial activity against both *S. aureus* and *Listeria*, which could indicate a certain ability to outcompete other intestinal taxa. 12B3-5 also harbours fewer pathogenic factors than other strains, since it is resistant only to rifampicin [14] and it is negative for the *esp* factor [14], in contrast to the significant trend observed for the strains isolated from high fat diet-fed mice [14].

Two strains were chosen as representatives of their respective STs to further pursue a proteomic study. The two proteomic files retrieved from the database were compared in a search of non-shared entries. A total of 280 were found; they were classified according to their putative functions found in the literature and their percentages with respect to the total unshared entries of each file, which were contrasted between both of them. Only in three categories did the figures in one strain exceed double those in the other: those related to cell structure, to bacteriophages (four folds higher) and to defence, with a higher percentage of 12B3-5 in all cases. The latter one seemed of special interest in a strain that had survived and overcome other competitors in a controlled environment. Among the 12B3-5 entries in this category, there were several peptides with antimicrobial activity like BacG protein [29], involved in the synthesis of the non-ribosomally synthesised dipeptide antibiotic produced by *Bacillus subtillis*; the *E. faecalis* macrolide-efflux protein A [30], able to regulate internal macrolide levels; or the PcfF protein, an accessory factor necessary to initiate the transfer of the tetracycline resistance plasmid pCF10 [31]. pCF10 is a member of a family of *E. faecalis* pheromone-inducible conjugative plasmids, which are determinants for ecological relations and strain communication in the gut environment, contributing to the GI tract competitive fitness [32]. In this sense, the sexual pheromone cAM373 [33] has been detected as well. 12B3-5 also has peptides like HAD and NUDIX hydrolases, with important roles in xenobiotic detoxification, protection from oxidative stress and protection from metabolite accumulation [34,35]. All these entries design a strain able to outcompete other possible rivals in an intestinal niche.

In summary, combining culture-dependent, metagenomic and proteomic data, our study shows, in a controlled murine model subjected to different diets, how two different sets of closely related *E. faecalis* isolates, framed in two distinct MLSTs, compete to thrive in their hosts. Despite the circumstance that in the murine group fed with standard diet, only ST9 is detected at any timepoint, while in the butter-enriched diet, only ST40 is isolated, the fact that both ST9 and ST40 are found in the other two groups at different timepoints, as well as the detection of different metagenomic sequences, is indicative of the start-with possibility for both of them to colonise any of the four groups. At the end of the experiment, isolates, all of them belonging to ST40, can only be found in one murine group, the one fed with butter. In addition, our metagenomic study indicates a total homogeneity in all the V3–V5 r16S sequences retrieved in this group at that timepoint, in coincidence with the ST40 V3–V5 sequence isolated then. These results are indicative of an original *E. faecalis* community with a certain complexity that ends up restricted to only one diet where a single genotype has been able to survive and displace its competitors. With the available data, it is not possible to ascertain the reasons why this has happened, but, as discussed above, higher bacteriocin and antibiotic production and greater protection from environmental factors such as xenobiotics, oxidative stress and metabolite accumulation could have made a difference with respect to its contenders. The present study shows that *E. faecalis* was present in a significantly higher percentage in the butter-enriched diet and uncovers the nature of the only strain that managed to thrive. Whether this is happening due to the fat nutritional characteristics, the strain’s arsenal of defensive proteins or a combination of both has yet to be dilucidated.

## 4. Materials and Methods 

### 4.1. Bacterial Isolates and Cultivation Conditions

A collection of 15 *E. faecalis* isolates from different diet-fed mice [14,36] were used for this study (Table 4). Briefly, as described previously [36], twelve male Swiss Webster ICR (CD-1) mice (Harlan laboratory) were divided into four groups and fed for twelve weeks with standard chow (SD; Panlab, Barcelona, Spain) or with chow supplemented with 20% of butter (BT), extra virgin olive oil (EVOO) or refined olive oil (ROO). All experimental procedures were approved by the Bioethics Committee of the University of Jaén, in accordance with the European Communities Council Directive 86/609/EEC. 

Putative enterococci were selected and characterised as also previously described [14]. Briefly, in that report, a large collection of bacterial strains isolated from the faeces of mice were screened for enterococcal characteristics and, after characterising and genotyping to avoid strain repetitions, 50 distinct enterococci belonging to several species were selected. The species *E. faecalis* was represented by 15 strains that are the subject of the present work (Table 1). Isolates were cultivated routinely on brain heart infusion (BHI) broth (Scharlab, Barcelona, Spain) at 37 °C and stored at −80 °C in BHI plus 20% glycerol.

### 4.2. Bacteriocin Production

Bacteriocin production in the strains was first tested in overnight cultures using the spot-on-a-lawn method on brain heart infusion agar, buffered with 0.15M sodium phosphate, pH 7.2 [37]. After 12 h of incubation at 37 °C, plates were overlaid with buffered soft agar previously inoculated with the indicator strains (*Listeria monocytogenes* CECT 4032, *Enterococcus faecalis* S47, *Staphylococcus aureus* CECT 976, *Escherichia coli* CECT 4972, *Salmonella cholerasuis* CECT 4300). Ten microliters of a 10 mg/mL trypsin solution were added on the side of each spot in order to prove the protein nature of the inhibitors. Plates were incubated for a further 3 h and then overlaid as above. All the culture supernatants of the strains that produced halos were further tested by the agar well diffusion method using stainless steel cylinders of 8 mm (outer) diameter [38]. 

### 4.3. MLST Analysis

The isolated strains were analysed by MLST. Briefly, specific PCRs were performed to check the presence of highly conserved small fragments of certain genes in the species *E. faecalis*, available at http://efaecalis.mlst.net/. The allelic profiles of isolates were obtained by sequencing internal fragments of seven housekeeping genes: gdh (glucose-6-phosphate dehydrogenase), gyd (glyceraldehyde-3-phosphate dehydrogenase), pstS (phosphate transporter for ATP binding), gky (glucokinase), aroE (siquimate-5-dehydrogenase), xpt (xanthine phosphoribosyltransferase) and yqiL (acetyl-CoAacetyltransferase). The PCR conditions for all amplification reactions were as follows: initial denaturation at 94 °C for 3 min; 35 cycles at 94 °C for 3 s, 50 °C for 3 s, and 72 °C for 3 s; and extension at 72 °C for 5 min. Reactions were performed in 50 µL volumes with buffers and Taq polymerase. The PCR products were purified with ExoSAP-IT (Applied Biosystems^TM^, Foster City, CA, USA) and sequenced with the respective primers in a CEQ 2000 XL DNA Analysis System (Beckman Coulter Inc., Fullerton, CA, USA). The sequences obtained were analysed in a built database with all the possible alleles for each gene with the BioEdit programme, aligning and comparing our sequence with the sequences of the database until we had an allele whose sequence was identical to the one obtained. Once we had the allele allocation, it was submitted to the *E. faecalis* MLST database (http://www.mlst.net) for the sequence type assignment. Cluster analysis of the data was performed using the MLST database and the eBURST algorithm. The sequence types of isolates are defined by the allelic profile at these seven loci, with each unique combination of alleles assigned a distinct sequence type number, and the sequence types (STs) of all isolates were determined.

### 4.4. r16S V3–V5 Sequence Identification and Analysis

The faecal bacterial community was studied by pyrosequencing the amplified metagenomic 16S rRNA as already described [11]. Nucleic acids were purified using QIAamp© DNA Stool Kit (QIAGEN), and sequencing was performed at Lifesequencing (Valencia, Spain) as described [11] after thirty-five libraries were constructed. Quality control (Q20 threshold) and checking for quimeras (UCHIME v. 4.2.40 programme) have also been detailed before [11]. Taxonomic levels were assigned through the Ribosomal Database Project Classifier. Statistically significant differences according to the type of diet were tested at the 5% level by Kruskal–Wallis analysis.

### 4.5. E. faecalis Protein Extraction

Overnight cultures in BHI were washed with 20 mM phosphate buffer pH 7.5 and resuspended in homogenisation buffer (50 mM TRIS buffer pH 7.6, 1 mM PMSF, 1 mM EDTA, 0.1% SDS, 2 mM DTT and one protease inhibitor tablet (Roche-11697498001) per 50 mL buffer). Crystal balls (Sigma-G9268) up to a volume equivalent to the pellet of each sample were added. Homogenates of the pellets were obtained by sonication (Bioruptor^®^, Center for Scientific-Technical Instrumentation of the University of Jaén, CICT) and supernatants were kept. Supernatant proteins were precipitated using the TCA–acetone protocol [39], and the precipitates obtained were dried in a laminar flow chamber and resuspended in 9 M urea, 4% CHAPS, 2% IPG buffer, 100 mM DTT and 0.001% bromophenol blue. Extracts were centrifuged at 13,000× *g* at 4 °C for 5 min, and the supernatants were collected. The protein profiles were checked by SDS-PAGE at the CICT.

### 4.6. 2-D Gel Electrophoresis

2-D protein extract analysis was carried out at the CICT following an adaptation of a previous report [40]. Protein extracts (125 µL) were loaded onto 7 cm immobilin strips, and the isoelectric focusing protocol was as follows: passive rehydration for 12 h at 20 °C (without pause); 50 V for 10 h (fast); 500 V for 1 h (fast); 1000 V for 1 h (fast); 4000 V for 30 min (linear); 400 V, 6000 V for 1 h (fast); and 500 V for 99 h (fast) (hold step), followed by separation in a 12% SDS–polyacrylamide gel as reported previously [40]. The gels were stained with Oriole (Bio-Rad, Hercules, CA, USA), and the image was captured with a Versadoc 4000 MP (Bio-Rad, Hercules, CA, USA). The gels were compared using the PDQuest^TM^ Analysis software (Bio-Rad, Hercules, CA, USA).

### 4.7. Analysis of Protein Extracts by Orbitrap Mass Spectrophotometry and Data Processing

The proteome analysis of the *E. faecalis* protein extracts by mass spectrometry and their corresponding data processing were carried out in a Thermo Scientific EASY-nLC 1000 nano-liquid chromatograph system/Thermo Q-Exactive Orbitrap mass spectrometer (Thermo Scientific, San Jose, CA, USA) at the CICT facilities of the University of Jaén in collaboration with the staff. For this, samples were digested in a solution following the protocol of the FASP protein digestion kit (Expedeon, Heidelberg, Germany) followed by a desalination according to the Pierce C18 Spin Columns protocol of Thermo Fisher Scientific. The results were compared with a specific database for *Enterococcus faecalis* in Uniprot.org with Proteome Discoverer 1.4 (Thermo Fisher Scientific, San Jose, CA, USA) and the search engine Sequest HT.

## Figures and Tables

**Table 1:** Alleles and multilocus sequence type adscription for each studied strain.

Strain	*gdh*	*gyd*	*pstS*	*gki*	*aroE*	*xpt*	*yqil*	ST	Closest BLAST
0S1-1	4	5	16	4	1	1	4	ST9	*E. faecalis* KB1
6S5-1	4	6	16	4	1	1	4	ST9	*E. faecalis* KB1
6S5-3	4	6	16	4	1	1	4	ST9	*E. faecalis* KB1
0B2-4	3	6	23	12	9	10	7	ST40	*E. faecalis* D32
6B1-2	3	6	23	12	1	14	7	ST150	*E. faecalis* D32
12B2-4	3	6	23	12	9	10	7	ST40	*E. faecalis* D32
12B3-4	3	6	23	12	9	10	7	ST40	*E. faecalis* D32
12B3-5	3	6	23	4	9	10	7	ST40	*E. faecalis* D32
0O5-4	3	6	23	12	9	10	nd	ST40	*E. faecalis* D32
0O7-3	4	6	16	4	1	1	4	ST9	*E. faecalis* KB1
6O5-3	3	6	23	12	9	10	7	ST40	*E. faecalis* D32
6O5-4	3	6	23	12	9	10	7	ST40	*E. faecalis* D32
6O6-1	3	6	23	12	9	10	7	ST40	*E. faecalis* D32
0V5-2	4	6	16	4	1	1	4	ST9	*E. faecalis* KB1

**Table 2:** Number of copies of 12B3-5 r16S V3-V5 sequence (in brackets) with respect to total *E. faecalis* copies in the metagenomic faecal sample data [8,11]. BT: butter enriched diet; OV: virgin olive oil enriched diet; ROO: refined enriched oil; SD: standard diet; W: weeks of experiment.

Mouse	BT_0w_	BT_12w_	OV_0w_	OV_12w_	ROO_0w_	ROO_12w_	SD_0w_	SD_12w_
1	0	0	n.d.	0	0	0	0	0
2	11 (7)	0	n.d.	0	0	0	0	0
3	0	7 (7)	n.d.	0	0	0	0	0
4	1 (0)	0	0	2 (0)	0	0	0	0
5	0	22 (22)	0	0	0	0	0	0
6	2 (0)	0	0	0	0	0	0	0
7	n.d.	1 (1)	0	0	0	0	0	0
8	n.d.	5 (5)	0	0	0	3 (0)	0	0
9	0	14 (14)	n.d.	0	0	0	n.d.	n.d.

n.d.: Not determined.

**Table 3:** Percentages of different types of non-shared proteins, present either in the 12B3-4 or the 0V5-2 proteomes, grouped according to their predicted roles.

Related to	12B3-5	0V5-2
Transport	13.2	10.1
Genetic material	19.8	28.8
ATP	2.2	2.0
Protein metabolism	4.4	7.1
Cell structure	4.4	1.5
Defence	15.4	7.1
Division	2.2	1.5
Bacteriophages	4.4	1.0
Others	14.3	22.7
Unknown	20.9	18.2

**Table 4:** Antibiotic resistance, virulence factors (enterococcal surface protein) and biogenic amine production (tyrosine decarboxylase) previously detected [14] in the 15 *E. faecalis* strains used in this study.

Strain	Number of Antibiotic Resistances Detected	*esp*	*tdc*
0S1-1	9	negative	negative
0B2-4	11	positive	negative
0O5-4	3	positive	positive
0O7-3	8	positive	negative
0V5-2	3	positive	positive
6S5-1	0	negative	positive
6S5-3	1	positive	positive
6B2-1	9	positive	positive
6O5-3	10	negative	positive
6O5-4	11	positive	positive
6O6-1	10	positive	positive
6V5-1	4	negative	positive
12B1-4	10	positive	positive
12B3-4	10	negative	positive
12B3-5	1	negative	positive

## Supplementary Materials

Supplementary materials can be found at https://www.mdpi.com/1422-0067/21/12/4330/s1.
