# Supplementary material for: Prevalence of an Intestinal ST40 Enterococcus faecalis over Other E. faecalis Strains in the Gut Environment of Mice Fed Different High Fat Diets
Source: Int J Mol Sci. 2020 Jun 18;21(12):4330. doi: 10.3390/ijms21124330 (PMC7352901; doi:10.3390/ijms21124330)
Supplement: Supplementary file 1 [file ijms-21-04330-s001.pdf]

**Supplementary material S1**. Entries detected in the proteomic *E. faecalis* Uniprot.org database after Orbitrap Mass Spectrometry which are not shared by both strains 12B3-5 and 0OV5-2.	
**Detected only in 12B3-5**	**Detected only in 0OV5-2**
	16S rRNA methyltransferase OS=Enterococcus faecalis GN=VS87_05325 PE=4 SV=1 - [A0A0L6YCR6_ENTFL]
	2-hydroxyglutaryl-CoA dehydratase OS=Enterococcus faecalis GN=VS87_01420 PE=4 SV=1 - [A0A0L6YEU8_ENTFL]
	23S rRNA methyltransferase OS=Enterococcus faecalis GN=VS87_09570 PE=4 SV=1 - [A0A0L6YC87_ENTFL]
	5-bromo-4-chloroindolyl phosphate hydrolysis protein OS=Enterococcus faecalis GN=VS87_08360 PE=4 SV=1 - [A0A0L6YC79_ENTFL]
	5-keto-4-deoxyuronate isomerase OS=Enterococcus faecalis GN=VS87_13080 PE=4 SV=1 - [A0A0L6Y9J5_ENTFL]
60 kDa chaperonin (Fragment) OS=Enterococcus faecalis GN=cpn60 PE=4 SV=1 - [D2WP86_ENTFL]	
ABC transporter (Fragment) OS=Enterococcus faecalis GN=efrA PE=3 SV=1 - [A0A060RQ19_ENTFL]	
ABC transporter ATP-binding protein OS=Enterococcus faecalis GN=ef0099 PE=3 SV=1 - [Q8KU62_ENTFL]	
ABC transporter OS=Enterococcus faecalis GN=abc1071 PE=3 SV=1 - [Q8GFE0_ENTFL]	
ABC transporter OS=Enterococcus faecalis GN=VS87_13755 PE=4 SV=1 - [A0A0L6Y9G6_ENTFL]	
ABC transporter permease OS=Enterococcus faecalis GN=VS87_03220 PE=4 SV=1 - [A0A0L6YFT5_ENTFL]	
	Acetyl-CoA carboxylase subunit alpha OS=Enterococcus faecalis GN=VS87_07800 PE=4 SV=1 - [A0A0L6YB88_ENTFL]
	Adenine methyltransferase OS=Enterococcus faecalis GN=VS87_04055 PE=4 SV=1 - [A0A0L6YG90_ENTFL]
	Adenylosuccinate lyase OS=Enterococcus faecalis GN=VS87_09780 PE=4 SV=1 - [A0A0L6YCD6_ENTFL]
	ADP-ribose pyrophosphatase OS=Enterococcus faecalis GN=VS87_08365 PE=4 SV=1 - [A0A0L6YD54_ENTFL]
	Aldehyde oxidoreductase OS=Enterococcus faecalis GN=VS87_08950 PE=4 SV=1 - [A0A0L6YBX9_ENTFL]
	Alpha-glycerophosphate oxidase OS=Enterococcus faecalis GN=VS87_03805 PE=4 SV=1 - [A0A0L6YGN9_ENTFL]
	Alpha/beta hydrolase OS=Enterococcus faecalis GN=VS87_11945 PE=4 SV=1 - [A0A0L6YAJ2_ENTFL]
Amidase OS=Enterococcus faecalis GN=VS87_06635 PE=4 SV=1 - [A0A0L6YDP6_ENTFL]	
Amidophosphoribosyltransferase OS=Enterococcus faecalis GN=VS87_03415 PE=4 SV=1 - [A0A0L6YFX1_ENTFL]	
	Amino acid dehydrogenase OS=Enterococcus faecalis GN=VS87_00335 PE=4 SV=1 - [A0A0L6YEF2_ENTFL]
	Aminodeoxychorismate lyase OS=Enterococcus faecalis GN=VS87_13735 PE=4 SV=1 – [A0A0L6Y931_ENTFL]
	Aminotransferase V OS=Enterococcus faecalis GN=VS87_12820 PE=4 SV=1 - [A0A0L6Y9R0_ENTFL]
	AraC family transcriptional regulator OS=Enterococcus faecalis GN=VS87_01565 PE=4 SV=1 - [A0A0L6YF45_ENTFL]
	Arginine repressor OS=Enterococcus faecalis GN=argR PE=3 SV=1 - [Q93K69_ENTFL]
	ArgR family transcriptional regulator OS=Enterococcus faecalis GN=VS87_05355 PE=4 SV=1 - [A0A0L6YCS1_ENTFL]
Aromatic ring hydroxylase OS=Enterococcus faecalis GN=VS87_01975 PE=4 SV=1 - [A0A0L6YFA7_ENTFL]	
	ArsR family transcriptional regulator OS=Enterococcus faecalis GN=pLG2-0032 PE=4 SV=1 - [E3WD80_ENTFL]

	ArsR family transcriptional regulator OS=Enterococcus faecalis GN=VS87_09475 PE=4 SV=1 - [A0A0L6YC96_ENTFL]
	Aspartate aminotransferase OS=Enterococcus faecalis GN=VS87_05090 PE=4 SV=1 - [A0A0L6YCN9_ENTFL]
	Aspartate aminotransferase OS=Enterococcus faecalis GN=VS87_05950 PE=4 SV=1 - [A0A0L6YD37_ENTFL]
	ATP synthase F0F1 subunit B OS=Enterococcus faecalis GN=VS87_08755 PE=4 SV=1 - [A0A0L6YCG9_ENTFL]
	ATP synthase F0F1 subunit delta OS=Enterococcus faecalis GN=VS87_08760 PE=4 SV=1 - [A0A0L6YC56_ENTFL]
	ATP synthase subunit J OS=Enterococcus faecalis GN=VS87_12720 PE=4 SV=1 - [A0A0L6Y9S9_ENTFL]
ATP synthase subunit K OS=Enterococcus faecalis GN=VS87_01890 PE=4 SV=1 - [A0A0L6YF92_ENTFL]	
	ATP-binding protein OS=Enterococcus faecalis GN=VS87_05705 PE=4 SV=1 - [A0A0L6YD65_ENTFL]
ATP-dependent helicase OS=Enterococcus faecalis GN=VS87_00595 PE=4 SV=1 - [A0A0L6YEK2_ENTFL]	ATP-dependent DNA helicase PcrA OS=Enterococcus faecalis GN=VS87_06710 PE=4 SV=1 - [A0A0L6YDR0_ENTFL]
	ATPase OS=Enterococcus faecalis GN=VS87_02045 PE=4 SV=1 - [A0A0L6YFR8_ENTFL]
ATPase OS=Enterococcus faecalis GN=VS87_09660 PE=4 SV=1 - [A0A0L6YCY3_ENTFL]	
ATPase V OS=Enterococcus faecalis GN=VS87_01900 PE=4 SV=1 - [A0A0L6YF34_ENTFL]	
	Bacitracin ABC transporter ATP-binding protein OS=Enterococcus faecalis GN=VS87_04385 PE=4 SV=1 - [A0A0L6YGF0_ENTFL]
	Bee1 OS=Enterococcus faecalis GN=bee1 PE=4 SV=1 - [Q20JV5_ENTFL]
BacG protein OS=Enterococcus faecalis GN=bacG PE=4 SV=1 - [O52969_ENTFL]	
Bacterial group 4 Ig-like protein OS=Enterococcus faecalis GN=VS87_03495 PE=4 SV=1 - [A0A0L6YGG4_ENTFL]	
	Biotin--acetyl-CoA-carboxylase ligase OS=Enterococcus faecalis GN=VS87_11080 PE=4 SV=1 - [A0A0L6YB71_ENTFL]
	Cadmium transporter OS=Enterococcus faecalis GN=VS87_01785 PE=4 SV=1 - [A0A0L6YF77_ENTFL]
C4-dicarboxylate ABC transporter OS=Enterococcus faecalis GN=VS87_11980 PE=4 SV=1 - [A0A0L6YA88_ENTFL]	
Capsid protein OS=Enterococcus faecalis GN=VS87_04190 PE=4 SV=1 - [A0A0L6YGB6_ENTFL]	
	Carbamoyl phosphate synthase small subunit OS=Enterococcus faecalis GN=VS87_03105 PE=4 SV=1 - [A0A0L6YG86_ENTFL]
	Carbohydrate diacid regulator OS=Enterococcus faecalis GN=VS87_08610 PE=4 SV=1 - [A0A0L6YC26_ENTFL]
	Cell division protein FtsJ OS=Enterococcus faecalis GN=VS87_05360 PE=4 SV=1 - [A0A0L6YCU0_ENTFL]
	Cell division protein FtsX OS=Enterococcus faecalis GN=VS87_03320 PE=4 SV=1 - [A0A0L6YG10_ENTFL]
Cell wall-associated hydrolase OS=Enterococcus faecalis GN=nlp PE=4 SV=1 - [A0A0E3MTX3_ENTFL]	
	Cold-shock protein OS=Enterococcus faecalis GN=VS87_03150 PE=4 SV=1 - [A0A0L6YFR7_ENTFL]
Cro/CI family transcriptional regulator OS=Enterococcus faecalis GN=VS87_08570 PE=4 SV=1 - [A0A0L6YBR9_ENTFL]	
	CylA OS=Enterococcus faecalis GN=cylA PE=3 SV=1 - [I2CZV9_ENTFL]
	CylR2 OS=Enterococcus faecalis GN=cylR2 PE=1 SV=1 - [Q8VL32_ENTFL]
D-alanine--D-alanine ligase OS=Enterococcus faecalis GN=vanG PE=1 SV=1 - [Q6WRY5_ENTFL]	

	DegV family EDD domain-containing protein OS=Enterococcus faecalis GN=VS87_11570 PE=4 SV=1 - [A0A0L6YA09_ENTFL]
	DeoR faimly transcriptional regulator OS=Enterococcus faecalis GN=VS87_11675 PE=4 SV=1 - [A0A0L6YB83_ENTFL]
	Dihydroorotase OS=Enterococcus faecalis GN=VS87_03110 PE=4 SV=1 - [A0A0L6YFW8_ENTFL]
	Dihydroorotate dehydrogenase OS=Enterococcus faecalis GN=dhoD1a PE=3 SV=1 - [Q841I8_ENTFL]
Dihydroorotate dehydrogenase OS=Enterococcus faecalis GN=VS87_12670 PE=4 SV=1 - [A0A0L6YA54_ENTFL]	
DNA mismatch repair protein MutL OS=Enterococcus faecalis GN=mutL PE=3 SV=1 - [S4TKL7_ENTFL]	DNA mismatch repair protein MutS OS=Enterococcus faecalis GN=mutS PE=3 SV=1 - [S4TKL8_ENTFL]
DNA mismatch repair protein MutS OS=Enterococcus faecalis GN=VS87_10765 PE=4 SV=1 - [A0A0L6YBK9_ENTFL]	DNA mismatch repair protein MutT OS=Enterococcus faecalis GN=VS87_02370 PE=4 SV=1 - [A0A0L6YFC1_ENTFL]
DNA polymerase III PolC-type OS=Enterococcus faecalis GN=polC PE=3 SV=1 - [Q8KT09_ENTFL]	
DNA polymerase III subunit delta OS=Enterococcus faecalis GN=VS87_09360 PE=4 SV=1 - [A0A0L6YCE3_ENTFL]	
DNA polymerase IV OS=Enterococcus faecalis GN=VS87_08100 PE=4 SV=1 - [A0A0L6YC21_ENTFL]	
	DNA topoisomerase I OS=Enterococcus faecalis GN=VS87_02650 PE=4 SV=1 - [A0A0L6YFH9_ENTFL]
DNA topoisomerase OS=Enterococcus faecalis PE=4 SV=1 - [E3WDA4_ENTFL]	
EF0117 OS=Enterococcus faecalis GN=ef0117 PE=4 SV=1 - [Q8KU46_ENTFL]	EF0129 OS=Enterococcus faecalis GN=ef0129 PE=4 SV=1 - [Q8KU35_ENTFL]
Ethanolamine utilization protein EutN OS=Enterococcus faecalis GN=VS87_02515 PE=4 SV=1 - [A0A0L6YFF6_ENTFL]	Ethanolamine utilization protein EutP OS=Enterococcus faecalis GN=VS87_02595 PE=4 SV=1 - [A0A0L6YFH0_ENTFL]
	Excinuclease ABC subunit A OS=Enterococcus faecalis GN=VS87_06510 PE=4 SV=1 - [A0A0L6YDM5_ENTFL]
Exodeoxyribonuclease III OS=Enterococcus faecalis GN=VS87_08195 PE=4 SV=1 - [A0A0L6YBL5_ENTFL]	Exodeoxyribonuclease VII large subunit OS=Enterococcus faecalis GN=VS87_05375 PE=4 SV=1 - [A0A0L6YCS7_ENTFL]
	Extracellular serine proteinase OS=Enterococcus faecalis GN=sprE PE=4 SV=1 - [A1YGU6_ENTFL]
Ferredoxin-NADP reductase OS=Enterococcus faecalis GN=VS87_13655 PE=4 SV=1 - [A0A0L6Y922_ENTFL]	
	Ferrous iron transporter A OS=Enterococcus faecalis GN=VS87_13310 PE=4 SV=1 - [A0A0L6Y9Z8_ENTFL]
	Filamentation induced by cAMP protein Fic OS=Enterococcus faecalis GN=pLG2-0013 PE=4 SV=1 - [Q9AL00_ENTFL]
FMN-binding protein OS=Enterococcus faecalis GN=VS87_11135 PE=4 SV=1 - [A0A0L6YB81_ENTFL]	
	General stress protein OS=Enterococcus faecalis GN=VS87_03245 PE=4 SV=1 - [A0A0L6YFU0_ENTFL]
Gluconate kinase OS=Enterococcus faecalis GN=VS87_11045 PE=4 SV=1 - [A0A0L6YB35_ENTFL]	
	Glucosamine-fructose-6-phosphate aminotransferase OS=Enterococcus faecalis GN=VS87_03900 PE=4 SV=1 - [A0A0L6YG71_ENTFL]
Glycerate kinase OS=Enterococcus faecalis GN=VS87_08605 PE=4 SV=1 - [A0A0L6YCE2_ENTFL]	
	Glycosyl transferase family 1 OS=Enterococcus faecalis GN=VS87_07725 PE=4 SV=1 - [A0A0L6YB72_ENTFL]
	Glycosyl transferase family 2 OS=Enterococcus faecalis GN=VS87_04720 PE=4 SV=1 - [A0A0L6YGK7_ENTFL]
GntR family transcriptional regulator OS=Enterococcus faecalis GN=VS87_03065 PE=4 SV=1 - [A0A0L6YFQ3_ENTFL]	
	Group II intron reverse transcriptase OS=Enterococcus faecalis GN=iep PE=4 SV=1 - [E1SKV0_ENTFL]

	GTPase OS=Enterococcus faecalis GN=VS87_07810 PE=4 SV=1 - [A0A0L6YBW2_ENTFL]
	Guanosine 5'-monophosphate oxidoreductase OS=Enterococcus faecalis GN=VS87_09445 PE=4 SV=1 - [A0A0L6YC63_ENTFL]
	HAD family hydrolase OS=Enterococcus faecalis GN=VS87_00720 PE=4 SV=1 - [A0A0L6YEH0_ENTFL]
	HAD family hydrolase OS=Enterococcus faecalis GN=VS87_07805 PE=4 SV=1 - [A0A0L6YBD7_ENTFL]
	HAD family hydrolase OS=Enterococcus faecalis GN=VS87_08440 PE=4 SV=1 - [A0A0L6YBQ1_ENTFL]
HAD family hydrolase OS=Enterococcus faecalis GN=VS87_12800 PE=4 SV=1 - [A0A0L6Y9F2_ENTFL]	
HAD superfamily hydrolase OS=Enterococcus faecalis GN=VS87_07825 PE=4 SV=1 - [A0A0L6YB93_ENTFL]	
	Heme ABC transporter ATP-binding protein OS=Enterococcus faecalis GN=VS87_04595 PE=4 SV=1 - [A0A0L6YGI7_ENTFL]
	Histidine kinase OS=Enterococcus faecalis GN=vanS PE=4 SV=1 - [Q0WYK9_ENTFL]
	Histidine kinase OS=Enterococcus faecalis GN=vanSL PE=4 SV=1 - [A9LN40_ENTFL]
	Holliday junction resolvase OS=Enterococcus faecalis GN=VS87_00795 PE=4 SV=1 - [A0A0L6YEI6_ENTFL]
	HrcA family transcriptional regulator OS=Enterococcus faecalis GN=VS87_01295 PE=4 SV=1 - [A0A0L6YET2_ENTFL]
	Hydroxylase OS=Enterococcus faecalis GN=VS87_08980 PE=4 SV=1 - [A0A0L6YC09_ENTFL]
	Hydroxymethylglutaryl-CoA synthase OS=Enterococcus faecalis GN=VS87_01595 PE=4 SV=1 - [A0A0L6YEZ4_ENTFL]
Hydroxymethylglutaryl-CoA synthase OS=Enterococcus faecalis GN=mvaS PE=1 SV=1 - [HMGCS_ENTFL]	
	Int410 OS=Enterococcus faecalis GN=int410 PE=4 SV=1 - [Q93A40_ENTFL]
	LacI family transcriptional regulator OS=Enterococcus faecalis GN=VS87_00975 PE=4 SV=1 - [A0A0L6YEN2_ENTFL]
	LacI family transcriptional regulator OS=Enterococcus faecalis GN=VS87_09900 PE=4 SV=1 - [A0A0L6YD23_ENTFL]
Lactate dehydrogenase OS=Enterococcus faecalis GN=VS87_14105 PE=4 SV=1 - [A0A0L6Y8Z4_ENTFL]	
	Leucyl-tRNA synthetase OS=Enterococcus faecalis GN=VS87_06325 PE=4 SV=1 - [A0A0L6YDY4_ENTFL]
	Lipoamidase OS=Enterococcus faecalis GN=lpa PE=4 SV=1 - [Q5XVM9_ENTFL]
Lipoprotein OS=Enterococcus faecalis GN=VS87_02405 PE=4 SV=1 - [A0A0L6YFJ1_ENTFL]	
	Lipoprotein OS=Enterococcus faecalis GN=VS87_08230 PE=4 SV=1 - [A0A0L6YBS2_ENTFL]
	Lipoprotein OS=Enterococcus faecalis GN=VS87_14055 PE=4 SV=1 - [A0A0L6Y9L2_ENTFL]
Macrolide ABC transporter ATP-binding protein OS=Enterococcus faecalis GN=VS87_01850 PE=4 SV=1 - [A0A0L6YGT2_ENTFL]	
Macrolide efflux protein A (Fragment) OS=Enterococcus faecalis GN=mefA PE=1 SV=1 - [MEFA_ENTFL]	
	MarR family transcriptional regulator OS=Enterococcus faecalis GN=VS87_08010 PE=4 SV=1 - [A0A0L6YBE8_ENTFL]
	MarR family transcriptional regulator OS=Enterococcus faecalis GN=VS87_12975 PE=4 SV=1 - [A0A0L6Y9T9_ENTFL]
	Membrane protein OS=Enterococcus faecalis GN=VS87_03450 PE=4 SV=1 - [A0A0L6YG25_ENTFL]

	MerR family transcriptional regulator OS=Enterococcus faecalis GN=VS87_03015 PE=4 SV=1 - [A0A0L6YFP2_ENTFL]
Membrane protein OS=Enterococcus faecalis GN=VS87_06465 PE=4 SV=1 - [A0A0L6YDF4_ENTFL]	
	Multidrug ABC transporter ATP-binding protein OS=Enterococcus faecalis GN=VS87_06870 PE=4 SV=1 - [A0A0L6YDM4_ENTFL]
	Multidrug ABC transporter ATP-binding protein OS=Enterococcus faecalis GN=VS87_13760 PE=4 SV=1 - [A0A0L6Y940_ENTFL]
	Multidrug ABC transporter permease OS=Enterococcus faecalis GN=VS87_06380 PE=4 SV=1 - [A0A0L6YDK2_ENTFL]
	N-acetylmannosamine-6-phosphate 2-epimerase OS=Enterococcus faecalis GN=VS87_11810 PE=4 SV=1 - [A0A0L6YA56_ENTFL]
	NAD(P)H nitroreductase OS=Enterococcus faecalis GN=VS87_12980 PE=4 SV=1 - [A0A0L6Y9H9_ENTFL]
	NADPH:quinone reductase OS=Enterococcus faecalis GN=VS87_11995 PE=4 SV=1 - [A0A0L6YAV5_ENTFL]
	Nucleoside hydrolase OS=Enterococcus faecalis GN=VS87_08870 PE=4 SV=1 - [A0A0L6YBW7_ENTFL]
NUDIX hydrolase OS=Enterococcus faecalis GN=VS87_00490 PE=4 SV=1 - [A0A0L6YEI2_ENTFL]	
	Ornithine carbamoyltransferase argF (Fragment) OS=Enterococcus faecalis PE=1 SV=1 - [Q7M182_ENTFL]
	Orotate phosphoribosyltransferase OS=Enterococcus faecalis GN=VS87_03080 PE=4 SV=1 - [A0A0L6YG82_ENTFL]
	Oxidoreductase OS=Enterococcus faecalis GN=VS87_00995 PE=4 SV=1 - [A0A0L6YEP6_ENTFL]
Oxidoreductase OS=Enterococcus faecalis GN=VS87_09735 PE=4 SV=1 - [A0A0L6YCE7_ENTFL]	
	PcfB OS=Enterococcus faecalis GN=pcfB PE=4 SV=2 - [Q5G3P0_ENTFL]
PBSX family phage terminase, large subunit OS=Enterococcus faecalis GN=VS87_04210 PE=4 SV=1 - [A0A0L6YGB9_ENTFL]	
PcfF OS=Enterococcus faecalis GN=pcfF PE=1 SV=1 - [Q5G3N6_ENTFL]	
Peptidase M20 OS=Enterococcus faecalis GN=VS87_12345 PE=4 SV=1 - [A0A0L6YB48_ENTFL]	Peptidase M23 OS=Enterococcus faecalis GN=VS87_04130 PE=4 SV=1 - [A0A0L6YGI2_ENTFL]
	Peptidase M4 OS=Enterococcus faecalis GN=VS87_01490 PE=4 SV=1 - [A0A0L6YEX4_ENTFL]
	Peptidase S9 OS=Enterococcus faecalis GN=VS87_01955 PE=4 SV=1 - [A0A0L6YGT1_ENTFL]
	Peptidase U32 OS=Enterococcus faecalis GN=VS87_11245 PE=4 SV=1 - [A0A0L6YBH1_ENTFL]
	Peptide ABC transporter substrate-binding protein OS=Enterococcus faecalis GN=VS87_10490 PE=4 SV=1 - [A0A0L6YB32_ENTFL]
	Peptide chain release factor 2 OS=Enterococcus faecalis GN=VS87_03330 PE=4 SV=1 - [A0A0L6YFV3_ENTFL]
	Phage infection protein (Fragment) OS=Enterococcus faecalis GN=pLG2-0069 PE=4 SV=1 - [F6KLM3_ENTFL]
	Phage tail protein OS=Enterococcus faecalis GN=VS87_04165 PE=4 SV=1 - [A0A0L6YGB2_ENTFL]
	Phenazine biosynthesis protein PhzF OS=Enterococcus faecalis GN=VS87_10050 PE=4 SV=1 - [A0A0L6YD49_ENTFL]
Phage protein OS=Enterococcus faecalis GN=VS87_01220 PE=4 SV=1 - [A0A0L6YFD5_ENTFL]	
	PhoP family transcriptional regulator OS=Enterococcus faecalis GN=VS87_00745 PE=4 SV=1 - [A0A0L6YEH6_ENTFL]
	PhoP family transcriptional regulator OS=Enterococcus faecalis GN=VS87_01065 PE=4 SV=1 - [H7C673_ENTFL]

	Phosphate ABC transporter ATP-binding protein OS=Enterococcus faecalis GN=VS87_03300 PE=4 SV=1 - [A0A0L6YFV2_ENTFL]
	Phosphate ABC transporter permease OS=Enterococcus faecalis GN=VS87_03305 PE=4 SV=1 - [A0A0L6YFU8_ENTFL]
Phosphate ABC transporter substrate-binding protein OS=Enterococcus faecalis GN=VS87_03315 PE=4 SV=1 - [A0A0L6YGC8_ENTFL]	
	Phosphoesterase OS=Enterococcus faecalis GN=VS87_11550 PE=4 SV=1 - [A0A0L6YAI7_ENTFL]
	Phosphoglycerate mutase OS=Enterococcus faecalis GN=VS87_13960 PE=4 SV=1 - [A0A0L6Y972_ENTFL]
	Phosphomethylpyrimidine kinase OS=Enterococcus faecalis GN=VS87_03980 PE=4 SV=1 - [A0A0L6YG79_ENTFL]
	Phosphomethylpyrimidine kinase OS=Enterococcus faecalis GN=VS87_08005 PE=4 SV=1 - [A0A0L6YBP0_ENTFL]
	Potassium transporter Trk OS=Enterococcus faecalis GN=VS87_13710 PE=4 SV=1 - [A0A0L6Y926_ENTFL]
Portal protein OS=Enterococcus faecalis GN=VS87_14125 PE=4 SV=1 - [A0A0L6Y932_ENTFL]	
	PrgW OS=Enterococcus faecalis GN=prgW PE=4 SV=1 - [Q51642_ENTFL]
Primosomal protein N OS=Enterococcus faecalis GN=VS87_10590 PE=4 SV=1 - [A0A0L6YB52_ENTFL]	
PTS beta-glucoside transporter subunit IIABC OS=Enterococcus faecalis GN=VS87_02455 PE=4 SV=1 - [A0A0L6YFZ3_ENTFL]	PTS beta-glucoside transporter subunit IIABC OS=Enterococcus faecalis GN=VS87_08820 PE=4 SV=1 - [A0A0L6YBV9_ENTFL]
	PTS fructose transporter subunit IIA OS=Enterococcus faecalis GN=VS87_13950 PE=4 SV=1 - [A0A0L6Y9W6_ENTFL]
	PTS fructose transporter subunit IIB OS=Enterococcus faecalis GN=VS87_02015 PE=4 SV=1 - [A0A0L6YF55_ENTFL]
PTS mannose transporter subunit IIC OS=Enterococcus faecalis GN=VS87_11595 PE=4 SV=1 - [A0A0L6YAA8_ENTFL]	PTS mannose transporter subunit IID OS=Enterococcus faecalis GN=VS87_03905 PE=4 SV=1 - [A0A0L6YH31_ENTFL]
Putative CRP/FNR transcriptional regulator OS=Enterococcus faecalis GN=arcR PE=4 SV=1 - [Q93K65_ENTFL]	
	Putative membrane arginine transporter OS=Enterococcus faecalis GN=arcD PE=4 SV=1 - [Q93K64_ENTFL]
	Putative resolvase OS=Enterococcus faecalis GN=resIP PE=4 SV=1 - [Q79A56_ENTFL]
	Putative transposase OS=Enterococcus faecalis GN=tnp PE=4 SV=1 - [Q47743_ENTFL]
	Putative uncharacterized protein OS=Enterococcus faecalis PE=4 SV=1 - [Q6WS10_ENTFL]
Putative uncharacterized protein OS=Enterococcus faecalis PE=4 SV=1 - [Q6WS11_ENTFL]	
	Queuine tRNA-ribosyltransferase OS=Enterococcus faecalis GN=tgt PE=4 SV=1 - [A0A0L6YD50_ENTFL]
Resolvase OS=Enterococcus faecalis GN=VS87_07690 PE=4 SV=1 - [A0A0L6YE75_ENTFL]	
	Resolvase OS=Enterococcus faecalis GN=res PE=4 SV=1 - [E1SKV7_ENTFL]
	Response regulator OS=Enterococcus faecalis PE=4 SV=1 - [H7C666_ENTFL]
	Restriction endonuclease OS=Enterococcus faecalis GN=pCPPF5_004 PE=4 SV=1 - [E1SKW2_ENTFL]
	Restriction endonuclease subunit S (Fragment) OS=Enterococcus faecalis GN=VS87_14550 PE=4 SV=1 - [A0A0L6Y960_ENTFL]
	Rhamnulokinase OS=Enterococcus faecalis GN=VS87_13115 PE=4 SV=1 - [A0A0L6Y9N2_ENTFL]

	Riboflavin biosynthesis protein RibD OS=Enterococcus faecalis GN=VS87_12715 PE=4 SV=1 - [A0A0L6Y9P3_ENTFL]
	Riboflavin biosynthesis protein RibF OS=Enterococcus faecalis GN=VS87_01240 PE=4 SV=1 - [A0A0L6YES4_ENTFL]
	Ribonuclease HIII OS=Enterococcus faecalis GN=VS87_01095 PE=4 SV=1 - [A0A0L6YEW2_ENTFL]
	Ribonuclease Z OS=Enterococcus faecalis GN=VS87_02985 PE=4 SV=1 - [A0A0L6YFU4_ENTFL]
	Ribonucleotide-diphosphate reductase subunit beta OS=Enterococcus faecalis GN=VS87_13290 PE=4 SV=1 - [A0A0L6Y9N6_ENTFL]
	RNA helicase OS=Enterococcus faecalis GN=VS87_05225 PE=4 SV=1 - [A0A0L6YCR5_ENTFL]
	RNA methyltransferase OS=Enterococcus faecalis GN=VS87_00545 PE=4 SV=1 - [A0A0L6YEJ2_ENTFL]
	RNA polymerase subunit sigma-54 OS=Enterococcus faecalis GN=VS87_10985 PE=4 SV=1 - [A0A0L6YB24_ENTFL]
	RNA-binding protein OS=Enterococcus faecalis GN=VS87_07815 PE=4 SV=1 - [A0A0L6YBK6_ENTFL]
	S-adenosylmethionine tRNA ribosyltransferase OS=Enterococcus faecalis GN=VS87_06055 PE=4 SV=1 - [A0A0L6YD56_ENTFL]
	SalA antigen (Fragment) OS=Enterococcus faecalis GN=salA PE=4 SV=1 - [E1ARY4_ENTFL]
	Serine kinase OS=Enterococcus faecalis GN=VS87_09490 PE=4 SV=1 - [A0A0L6YC86_ENTFL]
Sex pheromone cAM373 OS=Enterococcus faecalis GN=camE PE=4 SV=1 - [Q8KI26_ENTFL]	Sex pheromone cAD1 OS=Enterococcus faecalis GN=cad PE=4 SV=1 - [Q8RP94_ENTFL]
	Shikimate kinase OS=Enterococcus faecalis GN=VS87_02270 PE=4 SV=1 - [A0A0L6YFA0_ENTFL]
	Signal peptidase I OS=Enterococcus faecalis GN=VS87_00345 PE=4 SV=1 - [A0A0L6YE91_ENTFL]
	Single-stranded DNA-binding protein OS=Enterococcus faecalis GN=ef0030 PE=3 SV=1 - [Q8KUB6_ENTFL]
	Single-stranded DNA-binding protein OS=Enterococcus faecalis GN=pcfS PE=3 SV=1 - [Q5G3M3_ENTFL]
	Single-stranded DNA-binding protein OS=Enterococcus faecalis PE=3 SV=1 - [B6ZHL5_ENTFL]
Sortase OS=Enterococcus faecalis GN=VS87_10280 PE=4 SV=1 - [A0A0L6YAM9_ENTFL]	
Spermidine acetyltransferase OS=Enterococcus faecalis GN=VS87_00210 PE=4 SV=1 - [A0A0L6YE73_ENTFL]	
Spermidine/putrescine ABC transporter ATP-binding protein OS=Enterococcus faecalis GN=VS87_08575 PE=4 SV=1 - [A0A0L6YBT8_ENTFL]	
Srt2 OS=Enterococcus faecalis GN=srt2 PE=4 SV=1 - [Q20JV1_ENTFL]	Srt1 OS=Enterococcus faecalis GN=srt1 PE=4 SV=1 - [Q20JV2_ENTFL]
	Sugar ABC transporter substrate-binding protein OS=Enterococcus faecalis GN=VS87_09920 PE=4 SV=1 - [A0A0L6YCI2_ENTFL]
Sulfate ABC transporter ATP-binding protein OS=Enterococcus faecalis GN=VS87_01495 PE=4 SV=1 - [A0A0L6YEW1_ENTFL]	Sulfurtransferase OS=Enterococcus faecalis GN=VS87_07950 PE=4 SV=1 - [A0A0L6YBZ4_ENTFL]
Superoxide dismutase (Fragment) OS=Enterococcus faecalis GN=sodA PE=3 SV=1 - [G9FLV1_ENTFL]	Superoxide dismutase OS=Enterococcus faecalis GN=VS87_13260 PE=4 SV=1 - [A0A0L6Y9Y9_ENTFL]
Surface exclusion protein OS=Enterococcus faecalis GN=sea1 PE=4 SV=1 - [B6ZHR4_ENTFL]	Surface exclusion protein OS=Enterococcus faecalis GN=sea1 PE=4 SV=1 - [B6ZHR4_ENTFL]
	TetO OS=Enterococcus faecalis GN=tetO PE=4 SV=1 - [Q5GBH6_ENTFL]
	Tetr family transcriptional regulator OS=Enterococcus faecalis PE=4 SV=1 - [E3WDC7_ENTFL]
Threonyl-tRNA synthase OS=Enterococcus faecalis GN=VS87_07880 PE=4 SV=1 - [A0A0L6YCJ5_ENTFL]	
TraC protein OS=Enterococcus faecalis GN=EP0001 PE=4 SV=1 - [Q9F1J4_ENTFL]	
TraE protein OS=Enterococcus faecalis GN=EP0046 PE=4 SV=1 - [Q9F1F1_ENTFL]	

	Transcription-repair coupling factor OS=Enterococcus faecalis GN=VS87_12440 PE=4 SV=1 - [A0A0L6YAL3_ENTFL]
Transcriptional regulator OS=Enterococcus faecalis GN=VS87_05785 PE=4 SV=1 - [A0A0L6YD03_ENTFL]	Transcriptional regulator OS=Enterococcus faecalis GN=VS87_03785 PE=4 SV=1 - [A0A0L6YG51_ENTFL]
	Transcriptional regulator OS=Enterococcus faecalis GN=VS87_11210 PE=4 SV=1 - [A0A0L6YB96_ENTFL]
	Transglutaminase OS=Enterococcus faecalis GN=VS87_01985 PE=4 SV=1 - [A0A0L6YF54_ENTFL]
	Transglycosylase OS=Enterococcus faecalis GN=VS87_03215 PE=4 SV=1 - [A0A0L6YFZ0_ENTFL]
	tRNA (Guanine-N7)-methyltransferase OS=Enterococcus faecalis GN=VS87_06855 PE=4 SV=1 - [A0A0L6YE81_ENTFL]
	Tryptophanyl-tRNA synthetase OS=Enterococcus faecalis GN=VS87_08450 PE=4 SV=1 - [A0A0L6YCK0_ENTFL]
Tryptophan--tRNA ligase OS=Enterococcus faecalis GN=VS87_09950 PE=4 SV=1 - [A0A0L6YD31_ENTFL]	
Type I restriction enzyme R protein OS=Enterococcus faecalis GN=VS87_10070 PE=4 SV=1 - [A0A0L6YAK7_ENTFL]	
UDP-galactopuranose mutase OS=Enterococcus faecalis GN=VS87_09175 PE=4 SV=1 - [A0A0L6YCP0_ENTFL]	
	UDP-N-acetylglucosamine 1-carboxyvinyltransferase OS=Enterococcus faecalis GN=VS87_00630 PE=4 SV=1 - [A0A0L6YEE3_ENTFL]
	Uncharacterized protein OS=Enterococcus faecalis GN=pLG2-0030 PE=4 SV=1 - [F6KLP9_ENTFL]
	Uncharacterized protein OS=Enterococcus faecalis GN=VS87_00125 PE=4 SV=1 - [A0A0L6YEP0_ENTFL]
	Uncharacterized protein OS=Enterococcus faecalis GN=VS87_00195 PE=4 SV=1 - [A0A0L6YEC7_ENTFL]
	Uncharacterized protein OS=Enterococcus faecalis GN=VS87_00330 PE=4 SV=1 - [A0A0L6YES6_ENTFL]
	Uncharacterized protein OS=Enterococcus faecalis GN=VS87_00920 PE=4 SV=1 - [A0A0L6YEK4_ENTFL]
Uncharacterized protein OS=Enterococcus faecalis GN=VS87_00915 PE=4 SV=1 - [A0A0L6YEL7_ENTFL]	
	Uncharacterized protein OS=Enterococcus faecalis GN=VS87_01020 PE=4 SV=1 - [A0A0L6YGS2_ENTFL]
	Uncharacterized protein OS=Enterococcus faecalis GN=VS87_01255 PE=4 SV=1 - [A0A0L6YET6_ENTFL]
Uncharacterized protein OS=Enterococcus faecalis GN=VS87_01195 PE=4 SV=1 - [A0A0L6YEY0_ENTFL]	
	Uncharacterized protein OS=Enterococcus faecalis GN=VS87_01350 PE=4 SV=1 - [A0A0L6YEV0_ENTFL]
Uncharacterized protein OS=Enterococcus faecalis GN=VS87_01855 PE=4 SV=1 - [A0A0L6YF27_ENTFL]	
	Uncharacterized protein OS=Enterococcus faecalis GN=VS87_02115 PE=4 SV=1 - [A0A0L6YF74_ENTFL]
Uncharacterized protein OS=Enterococcus faecalis GN=VS87_02930 PE=4 SV=1 - [A0A0L6YG53_ENTFL]	
	Uncharacterized protein OS=Enterococcus faecalis GN=VS87_02975 PE=4 SV=1 - [A0A0L6YFM9_ENTFL]
	Uncharacterized protein OS=Enterococcus faecalis GN=VS87_03370 PE=4 SV=1 - [A0A0L6YGD9_ENTFL]
	Uncharacterized protein OS=Enterococcus faecalis GN=VS87_03935 PE=4 SV=1 - [A0A0L6YG74_ENTFL]
Uncharacterized protein OS=Enterococcus faecalis GN=VS87_04020 PE=4 SV=1 - [A0A0L6YGS5_ENTFL]	
	Uncharacterized protein OS=Enterococcus faecalis GN=VS87_04610 PE=4 SV=1 - [A0A0L6YGI6_ENTFL]
	Uncharacterized protein OS=Enterococcus faecalis GN=VS87_05205 PE=4 SV=1 - [A0A0L6YD81_ENTFL]
	Uncharacterized protein OS=Enterococcus faecalis GN=VS87_05280 PE=4 SV=1 - [A0A0L6YD92_ENTFL]
Uncharacterized protein OS=Enterococcus faecalis GN=VS87_04320 PE=4 SV=1 - [A0A0L6YGL2_ENTFL]	

Uncharacterized protein OS=Enterococcus faecalis GN=VS87_04565 PE=4 SV=1 - [A0A0L6YH12_ENTFL]	
Uncharacterized protein OS=Enterococcus faecalis GN=VS87_04610 PE=4 SV=1 - [A0A0L6YGI6_ENTFL]	
Uncharacterized protein OS=Enterococcus faecalis GN=VS87_04935 PE=4 SV=1 - [A0A0L6YH75_ENTFL]	
	Uncharacterized protein OS=Enterococcus faecalis GN=VS87_05975 PE=4 SV=1 - [A0A0L6YE35_ENTFL]
Uncharacterized protein OS=Enterococcus faecalis GN=VS87_06335 PE=4 SV=1 - [A0A0L6YDD4_ENTFL]	
	Uncharacterized protein OS=Enterococcus faecalis GN=VS87_06350 PE=4 SV=1 - [A0A0L6YDY9_ENTFL]
	Uncharacterized protein OS=Enterococcus faecalis GN=VS87_06770 PE=4 SV=1 - [A0A0L6YDK3_ENTFL]
	Uncharacterized protein OS=Enterococcus faecalis GN=VS87_06825 PE=4 SV=1 - [A0A0L6YDL4_ENTFL]
	Uncharacterized protein OS=Enterococcus faecalis GN=VS87_07130 PE=4 SV=1 - [A0A0L6YED2_ENTFL]
Uncharacterized protein OS=Enterococcus faecalis GN=VS87_07555 PE=4 SV=1 - [A0A0L6YEM6_ENTFL]	
	Uncharacterized protein OS=Enterococcus faecalis GN=VS87_09090 PE=4 SV=1 - [A0A0L6YC25_ENTFL]
Uncharacterized protein OS=Enterococcus faecalis GN=VS87_09295 PE=4 SV=1 - [A0A0L6YC64_ENTFL]	
	Uncharacterized protein OS=Enterococcus faecalis GN=VS87_10030 PE=4 SV=1 - [A0A0L6YCS8_ENTFL]
	Uncharacterized protein OS=Enterococcus faecalis GN=VS87_10045 PE=4 SV=1 - [A0A0L6YCK6_ENTFL]
Uncharacterized protein OS=Enterococcus faecalis GN=VS87_09710 PE=4 SV=1 - [A0A0L6YCZ2_ENTFL]	
Uncharacterized protein OS=Enterococcus faecalis GN=VS87_10015 PE=4 SV=1 - [A0A0L6YCI4_ENTFL]	
	Uncharacterized protein OS=Enterococcus faecalis GN=VS87_10610 PE=4 SV=1 - [A0A0L6YBI3_ENTFL]
	Uncharacterized protein OS=Enterococcus faecalis GN=VS87_11105 PE=4 SV=1 - [A0A0L6YB23_ENTFL]
	Uncharacterized protein OS=Enterococcus faecalis GN=VS87_11920 PE=4 SV=1 - [A0A0L6YAW4_ENTFL]
	Uncharacterized protein OS=Enterococcus faecalis GN=VS87_12455 PE=4 SV=1 - [A0A0L6YAJ1_ENTFL]
	Uncharacterized protein OS=Enterococcus faecalis PE=4 SV=1 - [A0A0E3MTF4_ENTFL]
	Uncharacterized protein OS=Enterococcus faecalis PE=4 SV=1 - [B1B1Y7_ENTFL]
Uncharacterized protein OS=Enterococcus faecalis PE=4 SV=1 - [A0A0E3KAU0_ENTFL]	
Uncharacterized protein OS=Enterococcus faecalis PE=4 SV=1 - [A0A0E3KI72_ENTFL]	
Uncharacterized protein OS=Enterococcus faecalis PE=4 SV=1 - [B6ZHM9_ENTFL]	
	Uncharacterized protein OS=Enterococcus faecalis PE=4 SV=1 - [G5CKM9_ENTFL]
	Uncharacterized protein OS=Enterococcus faecalis PE=4 SV=1 - [G5CKP1_ENTFL]
	Uncharacterized protein OS=Enterococcus faecalis PE=4 SV=1 - [K0FBY7_ENTFL]
	Universal stress protein UspA OS=Enterococcus faecalis GN=VS87_04045 PE=4 SV=1 - [A0A0L6YGS9_ENTFL]
	Uracil-DNA glycosylase OS=Enterococcus faecalis GN=VS87_05660 PE=4 SV=1 - [A0A0L6YDG3_ENTFL]
	Uridine kinase OS=Enterococcus faecalis GN=VS87_06260 PE=4 SV=1 - [A0A0L6YDC2_ENTFL]
	VE02 OS=Enterococcus faecalis PE=4 SV=1 - [C4P4H9_ENTFL]
	VE12 OS=Enterococcus faecalis PE=4 SV=1 - [C4P4I9_ENTFL]
VE19 OS=Enterococcus faecalis PE=4 SV=1 - [C4P4J6_ENTFL]	

	VigL protein (Fragment) OS=Enterococcus faecalis GN=vigL PE=4 SV=1 - [Q9REA5_ENTFL]
	von Willebrand factor protein OS=Enterococcus faecalis GN=VS87_00230 PE=4 SV=1 - [A0A0L6YE71_ENTFL]
VicR protein (Fragment) OS=Enterococcus faecalis GN=vicR PE=4 SV=1 - [Q9REA7_ENTFL]	
	YycI protein (Fragment) OS=Enterococcus faecalis GN=yycI PE=4 SV=1 - [Q9REA3_ENTFL]
